# Elements of Medical Jurisprudence

**Published:** 1860-03

**Authors:** 


### BOOK AND PAMPHLET NOTICES.

---

Elements of Medical Jurisprudence. By Theodoric Romeyn Beck, M. D., L. L. D., Professor of Materia Medica in the Albany Medical College. etc., etc., and John B. Beck, M. D., Professor of Materia Medica and Medical Jurisprudence in the College of Physicians and Surgeons of the City of New York, etc., etc. Eleventh Edition. With Notes by an Association of the friends of Dr. Beck. The whole revised by C. R. Gilman, M. D., Professor of Medical Jurisprudence in the College of Physicians and Surgeons of New York. In 2 vols., 8vo., pp. 884 and 1003. Philadelphia: J. B. Lippincott & Co. S. C. Griggs & Co, Chicago.

Medical Jurisprudence as defined, "is that science which applies the principles and practice of the different branches of medicine to the elucidation of doubtful questions in Courts of Justice." It is the administration of the law enlightened by medical knowledge. It is not the jurist sitting in judgment upon the medical man, but it is the latter aiding the former to discover the truth. And as in the ordinary exercise of our professional acquirements, the health and perhaps the life of the patient may depend upon the judgment and skill of the physician; so upon his testimony and knowledge may hang the fortune, the reputation, and even the life of the litigant. The physician is a welcome witness in every court, his opinion is sought with eagerness, listened to with respect, considered with care, and often in effect decides the merits of the case. How important then that the opinions of the medical expert should be enlightened and well founded. A verdict is seldom rendered against the clear, decided and unshaken testimony of scientific witnesses. As obvious as is the reliance of the jury upon the testimony of the medical witness, we are often chagrined to know that the character and standing of our profession is made a subject of ridicule, and brought into disrepute by the ignorance and unqualified pretensions of the medical witness. When we consider that the subject of medical jurisprudence is commensurate with medical knowledge, and that there is no time but that the same may be called into requisition to elucidate a litigated question. We hope that the time will never be again, when a student shall have attended three courses of lectures in any of our established Medical Schools, without having listened to more than *one* lecture upon this important branch. But until this subject is made universally a part of the curriculum in our schools, the inquiring student must depend upon the information to be gained from the study of the most approved text books and authoritative treatises. And we hazard but little in affirming our belief, that the work mentioned at the head of this notice is the best and most comprehensive treatise to be found in any language It has for many years occupied the foremost rank, and is so favorably known to our readers, that the announcement of this, the eleventh edition, is an all-sufficient recommendation. The previous character of former additions affords ample guarantee of continued improvements, and we have only to make a cursory examination of the volume before us, to be satisfied that the additions and addenda are both numerous and important The notes and inferences are selected with care and discrimination, and cannot fail to add materially to the practical value of the work.

Chapter 1st is devoted to Feigned Diseases, and chapter 2d to Disqualifying Disease; both have been carefully revised by R. H. Coolridge, M. D., Assistant Surgeon of the United States Army. Chapters 3, 4 and 5, have been prepared for the work by the careful and critical supervision of Austin Flint, M. D. Chapters 6 and 7, on Pregnancy and Delivery, have undergone a full revision by the aid of recently published facts and observations, by J. P. White, M. D.

The subject of Infanticide, by J. B. Beck, M. D., occupies a considerable portion of the 1st volume, and new cases are introduced illustrating the causes of death in new-born children. Also the history of Infanticide as it has prevailed in different nations; the murder of the fœtus in utero, with an account of the proofs and modes of perpetration; of the signs of Abortion, etc. The subjects of Legitimacy, Presumption of Survivorship, Age and Identity, Insurance upon Lives, respectively have been under the exclusive control of Prof. Gilman; and with that of Mental Aberration, by D. Tilden Brown, M. D., complete the first volume. The second volume contains a full chapter upon Persons Found Dead, upon Wounds of the Living Body, by John Watson, M. D. The most important part of the whole work is taken up in the consideration of the subject of Poisons, in which we observe that numerous cases have been added, including new facts regarding the fatal doses of some of these agents, and the pathological changes they produce; also the various improvements in the mode of applying tests for the detection of these agents, have been fully considered. The chapter on Medical Evidence is the last of the series, and while it is a subject that lies at the foundation of success in every department of medical inquiry, yet there is no way, as we have before suggested, in which the capacity of the medical man is so fully tested. A close attention to the suggestions contained in this chapter will prove interesting to the physician of experience, and particularly instructive to the young man who may desire to lay the surest foundation of a professional reputation. In short, those who are familiar with the character of the work in question, will be struck with many minor, but still useful changes, not mentioned in the preface. The index has been much extended and rendered more complete, and the execution of the work is neatly and carefully done: and we are satisfied that it will not only prove a manual comprehensive in detail, but will possess the weight and accuracy, as well as the fulness of an authoritative treatise.

